# Comparative analysis of the time-dependent functional and molecular changes in spinal cord degeneration induced by the *G93A SOD1 gene *mutation and by mechanical compression

**DOI:** 10.1186/1471-2164-9-500

**Published:** 2008-10-23

**Authors:** Andrea Malaspina, Natasa Jokic, Wenlong L Huang, John V Priestley

**Affiliations:** 1 Neuroscience Centre, Institute of Cell and Molecular Science, Barts and The London School of Medicine and Dentistry, Queen Mary University of London, 4 Newark Street, Whitechapel, London E1 2AT, UK

## Abstract

**Background:**

Mutations of the *superoxide dismutase 1 (SOD1) *gene are linked to amyotrophic lateral sclerosis (ALS), an invariably fatal neurological condition involving cortico-spinal degeneration. Mechanical injury can also determine spinal cord degeneration and act as a risk factor for the development of ALS.

**Results:**

We have performed a comparative ontological analysis of the gene expression profiles of thoracic cord samples from rats carrying the *G93A SOD1 gene *mutation and from wild-type littermates subjected to mechanical compression of the spinal cord. Common molecular responses and gene expression changes unique to each experimental paradigm were evaluated against the functional development of each animal model. Gene Ontology categories crucial to protein folding, extracellular matrix and axonal formation underwent early activation in both experimental paradigms, but decreased significantly in the spinal cord from animals recovering from injury after 7 days and from the *G93A SOD1 *mutant rats at end-stage disease. Functional improvement after compression coincided with a massive up-regulation of growth-promoting gene categories including factors involved in angiogenesis and transcription, overcoming the more transitory surge of pro-apoptotic components and cell-cycle genes. The cord from *G93A SOD1 *mutants showed persistent over-expression of apoptotic and stress molecules with fewer neurorestorative signals, while functional deterioration was ongoing.

**Conclusion:**

this study illustrates how cytoskeletal protein metabolism is central to trauma and genetically-induced spinal cord degeneration and elucidates the main molecular events accompanying functional recovery or decline in two different animal models of spinal cord degeneration.

## Background

Spinal cord degeneration in humans resulting from a mechanical injury or developing in the context of a neurodegenerative disorder causes a variable degree of neurological disability. Functional recovery after a mechanical insult to the spinal cord depends on the force and site of impact, on the duration of uninterrupted compression and possibly, on a genetically-determined ability of the nervous tissue to activate repair mechanisms. Similarly, non-traumatic neurodegenerative disorders affecting the spinal cord present a wide range of clinical variability. Amyotrophic lateral sclerosis/motor neuron disease (ALS/MND), for example, leads almost invariably to death from respiratory failure on average in 3 to 5 years from disease onset, as a result of widespread motor cell loss. However, survival rates of up to 15 years have been described, along with ALS cases who survive no more than 6 months [1]. Clinical heterogeneity has also been described in those 20% of familial ALS cases linked to mutations of the superoxide dismutase 1 *(SOD1) *gene, a genetic defect that exhibits a toxic gain of function that adversely affects motor neurons [2-4]. The molecular determinants that modulate the course of trauma-induced and genetically-induced spinal cord degeneration are still largely elusive. Spinal cord vulnerability may be linked to disease modifiers that are activated by selective disease processes but also depends on a common ground of molecular factors that can be switched on by different pathological triggers. Recent epidemiological observations strengthen the case for the search for molecular mechanism of spinal cord tissue degeneration that may be activated by different pathological determinants. Cervical trauma has been singled out as one of the main risk factors for the development of ALS and this deadly neurological disorder seems to be seven times more prevalent in a large cohort of professional footballers in Italy [5].

How biomechanical stress initiates the cascade of molecular events leading to the irreversible neurodegeneration observed in ALS is not known. We can postulate that the impact may accelerate a pathological process already ongoing or precipitate a detrimental tissue response that ultimately triggers the disease in genetically susceptible individuals. Novel techniques of gene expression and proteomic analysis have been applied to obtain a better understanding of the molecular events underlying trauma or genetically-induced degeneration. In spinal cord from animal models of spinal cord injury (SCI) and ALS, similar to what is observed in post-mortem spinal cord from ALS individuals, a time-dependent and coordinated expression of functionally synergistic gene candidates has been reported. This includes an early differential expression of components of the cytoskeleton, of the neurotransmission machinery and of genes involved in lipid metabolism, protein kinase regulation, antioxidant activity, lysosomal function, ion transport, transcription and inflammation [6-8]. 24 hours from mechanical trauma, the gene expression profile in spinal cord switches towards the activation of growth, axonal guidance, angiogenic, cell cycle and extracellular matrix factors while activated macrophages accumulate in the damaged tissue [9-13]. The ALS spinal cord molecular profile after disease onset shows the differential regulation of a wide range of inflammatory and stress-related molecular factors and of genes involved in zinc/copper and cholesterol homeostasis [7,14,15]. The role of these molecular pathways in preserving and restoring tissue functional integrity is not entirely clear. For example, in both SCI and in ALS animal models, the activation of inflammatory genes has been described in affected tissues but we do not know whether these responses have a detrimental effect on tissue regeneration [16,10,7,11,14,15]. A consensus exists about the effects of other trauma-induced gene expression changes, such as the post-injury secretion from activated astrocytes of anti-regenerative molecules like chondroitin sulphate proteoglycans and of the pro-regenerative factors laminin and fibronectin. The balance between these factors conditions gial scarring, which limits axonal regeneration [17]. Despite the wealth of data available, no study has attempted to amalgamate different experimental conditions to evaluate the role of specific molecular pathways in the context of functional recovery or decline, or to investigate the molecular interplay between trauma-induced and idiopathic degeneration.

In this study, we have analysed the biological significance of the temporal gene expression changes occurring in spinal cord degeneration induced by mechanical injury or determined by the *G93A SOD1 *gene mutation. We have used a bioinformatics approach (High-Throughput GoMiner) which allows the integration of different gene expression datasets, the dissection of Gene Ontology (GO) categories containing gene candidates sharing a similar functional role from the most significantly differentially expressed genes, and a comparative evaluation of the significance of these gene categories. To minimise differences inherent to the genetic background of the animal models, we have compared Sprague Dawley rats carrying the *G93A SOD1 *gene mutation with their wild type littermates after spinal cord compression injury. Longitudinal gene expression analyses were obtained at different time points within a week from spinal cord compression injury and at a pre-symptomatic stage, at disease onset and end-stage in the *G93A SOD1 *mutant rats. In these time-windows, both experimental paradigms of spinal cord degeneration display a remarkable degree of motor cell loss, in association with the development of a significant loco-motor improvement in the rat model of spinal cord compression (SCC) and of an irreversible functional decline in the rat model of ALS. The integrative analysis of a large group of molecular pathways acting within situations of acute and chronic degeneration that lead to completely different functional outcomes, provides a better understanding of the role of these molecular cascades in neurodegeneration. We have chosen the 30 minutes, 4 hours, 24 hours and 7 days time points for our experimental observations after SCC, to allow direct comparison with previous gene expression data generated by a similar investigation into the effects of a mild spinal cord injury [11] and with published histological data on the rat SCC model [18]. This study aims at advancing our understanding of spinal cord degeneration in two major areas: a) the identification of those molecular changes induced by mechanical trauma that may set the course of irreversible degeneration as seen in ALS, b) the main driving forces behind functional decline and recovery in genetically-induced and traumatic spinal cord degeneration.

## Results and discussion

### Behavioural and locomotor changes in the rat models of spinal cord compression and amyotrophic lateral sclerosis: histopathological correlations

**Figure 1:**
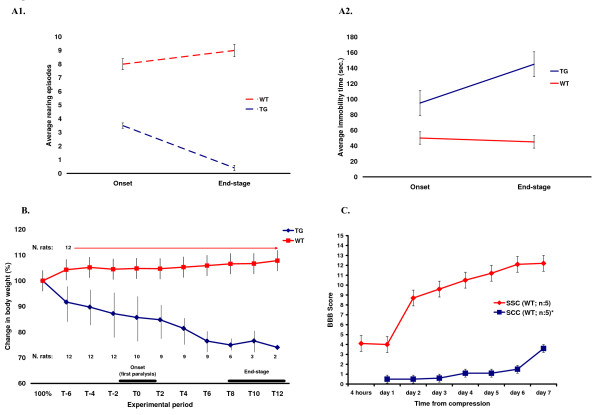
**Graphs illustrating the profiles of functional, behavioural and weight changes recorded in the models of trauma and genetically-induced spinal cord degeneration employed in this study.** The functional and behavioural analyses include the open-field locomotor assessment (BBB score) of rats after mild spinal cord compression and the evaluation of immobility time and rearing episodes in *G93A SOD1 *mutant rats. For comparison, we report previously published data generated from similar experimental platforms (data taken from Jokic et al. 2007 and Huang et al. 2007) [16,18]. **A1 **– **A2**: Schematic representation of the average immobility time (A2) and average number of rearing episodes (A1) observed in 4 *G93A SOD1 *mutant rats with subtle signs of hindlimb paralysis at disease presentation and in 4 wild type rats. Determinations by independent observers were performed at onset and at end-stage disease, based on 2 subsequent 3-minutes video clips recorded during disease development in the 4 *G93A SOD1 *mutant rats and in the WT littermates. Data previously reported by Jokic et al. [16]. At disease onset, the duration of average immobility time in the wild-type and in the *G93A SOD1 *mutant animals was similar, whereas a significant increase in duration of immobility time was observed in the *G93A SOD1 *mutant animals at end stage disease (A2; p < 0.05, ANOVA). A remarkable reduction of rearing episodes is seen in *G93A SOD1 *mutant rats at end-stage disease (A1; p < 0.001, ANOVA). Disease onset (appearance of first signs of paralysis): 129 +/- 17.3 days. End-stage disease (25–30% loss of body weight): 139 +/- 17 days. **B**: Individual body weight measurements of 12 *G93A SOD1 *mutant rats and of 12 age-matched wild type animals from a pre-symptomatic stage to end-stage disease (*G93A SOD1 *mutant rats presented with either hindlimb or forelimb paralysis at disease onset). The data presented include previous observations obtained from a group of 4 *G93A SOD1 *mutant rats and 4 wild type rats [16]. The maximum body weight recorded for each *G93A SOD1 *mutant rat was set at 100% as base-line. The graph indicates body weight measurements performed after this point (the measurements started at 7 weeks of age and were performed every two days), expressed as percentage of the maximum body weight. Disease onset (T0) is indicated at the time of the appearance of the first signs of paralysis (on average at 129 +/- 17.3 days of age). At disease onset, weight loss has been progressing for at least 6 days to a 10 to 15% reduction from maximum body weight. The end-stage disease was set at 25–30% of weight loss (139 +/- 17 days of age in the *G93A SOD1 *mutants). Weight measurements are expressed as averages of weight determinations of the number of surviving animals reported in the graph (those rats reaching a 25–30% body weight reduction were sacrificed). The graph reports the numbers of surviving animals at each time point. **C**: the graph illustrates the functional recovery of wild-type female rats after spinal cord compression, measured at 30 minutes, 4 hours, 24 hours and 7 days from mild injury (35 g weight spinal cord compression) using the Basso, Beattie, and Bresnahan (BBB) open-field locomotor assessment. The BBB scoring for each 5 animals right and left hindlimb was graded at the defined time points by two independent blinded observers (red colour code). Also plotted are the BBB scores of Sprague-Dawley rats after a 50 g compression injury (blue colour code, n: 5), taken from previously reported data [18].

The Basso, Beattie, and Bresnahan (BBB) open-field locomotor rating scale was applied to evaluate functional recovery after spinal cord compression (SCC). Figure 1C displays the curve of functional recovery according to BBB assessment in spinal cord compression (SCC) performed using 35 g. weight (red colour code; 0 represents hindlimb complete paralysis, 15 represents full weight support and forelimb/hindlimb coordination but incomplete toe clearance, 21 represents normal function) [18]. To demonstrate the effects of spinal cord compression (SCC) induced by a heavier weight, we have plotted in the same graph the previously reported data obtained using a 50 g compression injury model [18]. In rats subjected to a 35 g SCC, the Basso, Beattie, and Bresnahan (BBB) scoring showed the highest level of post-injury disability in the period between 4 hours and 24 hours after mechanical compression, followed by a steady recovery on day 2 and by a more gradual improvement to the final measurement at day 7 after injury.

Serial weight measurements of the *G93A SOD1 *mutant rats were performed from 7 weeks of age and the maximum body weight in pre-symptomatic rats was recorded. As already described [16], the body weight of the *G93A SOD1 *mutant rats is reduced by up to 10% of their maximum body weight when the first signs of paralysis become detectable (disease onset: 129 +/- 17.3 days of age; Figure 1B, T0), on average 6 days after the measurement of the maximum body weight (Figure 1B). Progression to the final stage of the disease (defined as weight reduction of 25% from maximum body weight), was very rapid and it was complete in approximately 7 days from disease onset (disease end-stage: 139 +/- 17 days of age; Figure 1B). Figure 1(A1, A2) reports previously published data obtained from a behavioural analysis of the *G93A SOD1 *mutant rats, outlining the increase of immobility time already evident at onset of the first symptoms accompanied by a reduction of rearing episodes. Late-stage disease in mutant animals is characterised by the significant increase of immobility time and by the reduction of rearing episodes [16].

It is possible to correlate the changes involving functional and behavioural aspects of the disease development in the animal models employed in this study, with the main pathological changes that have been previously described in the spinal cord from these models of neurodegeneration [16,18]. For example, the initial subtle signs of disease accompanied by a substantial weight loss in the *G93A SOD1 *mutant rat are associated with an already established reduction of the motor cell population, glial activation, axonal loss, infiltration of inflammatory cells and derangement of the intracellular architecture with the appearance of inclusion bodies [16,38]. While minor elements of tissue structural alteration are already present in an early pre-symptomatic stage, the pace of spinal cord tissue degeneration in the *G93A SOD1 *mutant rat accelerates from the onset to the advanced stage of the disease, in concert with the dramatic functional decline and weight loss described in this transgenic model of ALS (Figure 1 A1/A2/B).

Likewise, the functional effects of a 50 g spinal cord compression have been thoroughly described, together with the main pathological features of spinal cord degeneration developing in this type of mechanical injury [16]. Studies of spinal cord injury using static compression have highlighted that neuronal loss is much more protracted in time in this type of trauma, than the loss observed after contusion injury. While neuronal death following contusion is an acute event that is largely over after 24 hours, loss of neuronal cells after compression injury at the injury epicentre involves approximately 43% in the first day and extends to 73% of the whole neuronal cell population at 3 days after the impact. In this time-window, glial cells are also likely to become involved and the spinal cord tissue pathology is dominated by inflammatory responses including macrophage activation. The massive neuronal loss and the effects that mechanical compression exerts on cortico-spinal function explain the significant locomotor impairment in our animal model of compression injury in the first day post-injury. The much slower pace of neuronal loss from the second day after the trauma, together with the activation of a number of tissue-repair mechanism as reported later on in this paper, gives a reason for the remarkable functional recovery observed after the first day, particularly between 24 and 48 hours in the post-injury time-frame.

### Comparative gene ontology analysis of the time-dependent spinal cord molecular profiles induced by compression and by the G93A SOD1 gene mutation

**Figure 2:**
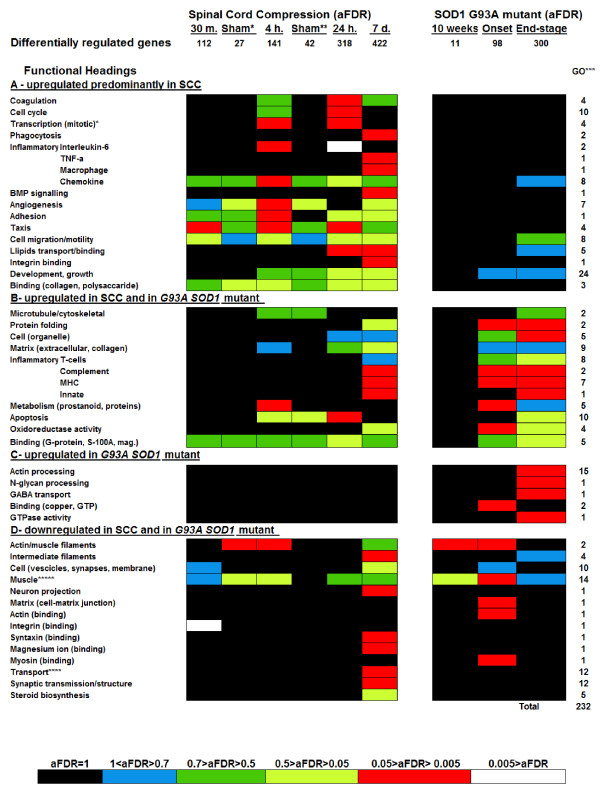
**Comparative ontological characterisation of the time-dependent spinal cord gene expression profiles obtained from animal models of spinal cord compression and *G93A SOD1 *mutant rats using Bead array technology (Illumina) and High-Throughput GoMiner.** The heat chart reports the main functional headings containing clusters of parent-child and functionally related Gene ontology (GO) categories (total output from High-Throughput GoMiner: 232 GO categories) obtained from the 9 subsets of differentially expressed genes. The 9 subsets of differentially regulated genes have been obtained comparing thoracic cord at 30 minutes, 4 hours, 24 hours and 7 days after compression injury and at 30 minutes and 4 hours after laminectomy with healthy 10-weeks old spinal cord, and comparing the *G93A SOD1 *mutant spinal cord from animals at a pre-symptomatic stage, at onset and at end-stage disease with spinal cord from wild-type littermates at pre-symptomatic, onset and end-stage disease time-points. GO categories are computed from the 9 text files containing the changed genes by High-Throughput GoMiner, according to estimations of significance by false discovery rate (FDR, cut off <0.05). The functional headings contain GO categories identified as significantly up-regulated predominantly in the spinal cord compression molecular profiles (A), in both *G93A SOD1 *mutant and injured spinal cord (B), and only in the spinal cord from the *G93A SOD1 *mutant animals (C). Functional headings down-regulated in spinal cord from both experimental conditions are also reported (D). The degree of differential regulation of each functional heading is expressed at a specific time point as the average of the false discovery rates (aFDR) of all the functional heading's GO categories at this specific time-point and represented in the heat-chart with a colour code, in which lighter colours represent a higher level of differential regulation and the darker ones a lower level of differential regulation. "Differentially regulated": number of genes differentially regulated found in spinal cord gene expression analysis at each time point in the two experimental coditions. Sham*: 30 minutes after sham operation. Sham**: 4 hours after sham operation. GO***: number of GO categories in each functional heading. ****: transport of ions, neurotransmitters, potassium, calcium. *****: striated muscle, sarcomere, myosin, thick filaments.

We have compared the thoracic cord molecular profiles of the *G93A SOD1 *mutant spinal cord obtained from animals at a pre-symptomatic, at onset and at end-stage disease, to the thoracic cord molecular profiles after spinal cord compression (SCC) and sham-operation at 30 minutes and 4 hours, and after spinal cord compression (SCC) at 24 hours and 7 days from the trauma. We have used the Illumina Bead-arrays (Illumina, San Diego, USA) to generate a total of 12 gene expression datasets from thoracic spinal cord samples, harvested from the above animal models at the specified time-points. These include the expression profiles of pooled thoracic cord samples at 30 minutes, 4 hours, 24 hours and 7 days from injury, at 30 minutes and 4 hours from sham-operation and the expression profile of pooled *SOD1 G93A *mutant thoracic cord samples and of pooled wild type (WT) littermates at a pre-symptomatic stage (10 weeks of age), at onset and at end-stage disease. Differential gene expression analysis was obtained comparing the expression profiles of the injured, sham-operated and *SOD1 G93A *mutant spinal cord pools with wild type (WT) pooled spinal cord tissues at the specified time points (the expression profile of the 10-week old WT thoracic cord was used as control to identify differential expression in injured spinal cord; numbers of differentially-regulated genes in thoracic spinal cord : 11 in pre-symptomatic *G93A SOD1 *mutant, 98 in disease onset *G93A SOD1 *mutant, 300 in disease end-stage *G93A SOD1*mutant; 112 genes in thoracic cord 30 minutes after compression, 27 genes in thoracic cord 30 minutes after laminectomy; 141 genes in thoracic cord 4 hours after compression; 42 genes in thoracic cord 4 hours after laminectomy; 318 genes in thoracic cord 24 hours after compression; 422 genes in thoracic cord 7 days after compression, Figure 2; "differentially regulated genes") and 9 lists of differentially-regulated gene candidates were generated and used for further ontological analysis using High-throughput GoMiner. A separate Bead-chip was used for the gene expression analysis of pools of lumbar spinal cord samples from *G93A SOD1 *mutant rats at a pre-symptomatic stage, at onset and at end-stage disease and from wild type littermates.

We have used High-Throughput GoMiner  to integrate the spinal cord gene expression data into a coherent picture of biological processes. This program provides a biological interpretation for the lists of under- and over-expressed genes generated by a microarray experiment, clustering functionally synergistic genes into Gene Ontology (GO) categories. The software defines those GO categories which are more represented within the total of differentially expressed genes according to a gene enrichment principle (number of genes of a specific GO category in the pool of differentially regulated genes) and according to a false discovery rate (FDR).

High-throughput GoMiner analysis of the 9 text files enlisting the changed genes generated a total of 232 GO categories with a significant differential regulation at one or more time points in the two experimental conditions, including 166 up-regulated and 66 down-regulated GO categories (FDR < 0.05). The 232 GO categories were grouped under specific functional headings, according to their parent-child relationship as well as their functional and biological affinity (Figure 2; "Functional Headings"; GO***: number of GO categories in each functional heading).

**Figure 3:**
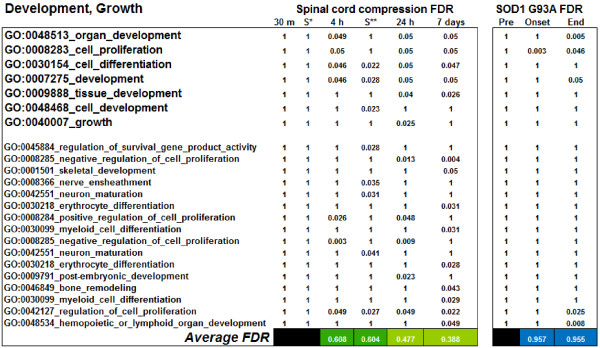
**Details of the GO categories composition of the functional heading "Development, growth" (23 GO categories, see also Figure 2A).** The underlined GO categories at the top of the graph are the parent categories, which have a broad functional role. The bottom of the graph reports the children categories, with fewer genes and more defined functional roles in the development of specific cell types (these genes are represented also in the parent categories). In both the compression and the *G93A SOD1 *mutant spinal cord, the selected GO categories FDRs are represented and the average of the FDR values for all the GO categories at each time points are represented at the bottom row with the colour code used in Figure 2.

To illustrate the level of differential regulation of each functional heading at a specific experimental time point for the SCC and the *G93A SOD1 *mutant spinal cords, we have calculated the average of the FDR values (aFDR) of the GO categories included in each functional heading at all time points considered in the two experimental conditions. Averages at each time point were obtained from both significant FDRs (< 0.05) and non significant FDRs (>0.05 – non-significant FDR were given the value of 1), computed for the GO categories included in each functional heading (Additional files 1, 2, 3 and 4 report the GO categories and FDRs included in the functional headings displayed in Figure 2A, B, C and 2D). Average FDRs (aFDR) were displayed in a heat chart with a colour code scale, the lighter colours representing a higher level and the darker colours a lower level of average statistical significance (Figure 2). Each aFDR colour in the heat-chart covers a range of FDR values, expressing the standard error of each calculation (Figure 2). As an example, Figure 3 provides information on the "Development, Growth" functional heading, its content in GO categories and the aFDRs calculated for each time points in the SCC and ALS experiments. Figure 2 displays functional headings whose GO categories are up-regulated predominantly in SCC (A; some headings are also represented in the *SOD1 G93A *mutant spinal cord), in both SCC and SOD1 G93A mutant cord samples (B), only in the *SOD1 G93A *mutant cord samples (C) or down-regulated in both SCC and *SOD1 G93A *mutant cord samples (D; see Additional files 1, 2, 3 and 4 respectively).

The GO categories and functional headings found to be significantly activated in sham-operated animals at 30 minutes and 4 hours were also differentially regulated in spinal cord from injured animals, including GO categories for chemokines, angiogenesis, taxis, cell migration/motility, growth/differentiation and microtubule formation (Figure 2). However, the difference in aFDR values of the functional headings common to injured and sham-operated spinal cord samples, demonstrated that gene expression changes were clearly more significant in the injured spinal cord. While the level of "Development, growth" functional heading activation at 4 hours is similar in injured and sham-operated animals, this functional heading's GO categories become more significantly up-regulated in injured spinal cord at 24 hours and 7 days from injury (Figure 2A). These data are in line with the previous observations of an early activation of a similar profile of molecular pathways, following both direct trauma to the spinal cord and sham operation [12]. The stress induced by simple laminectomy can have an effect on spinal cord tissue which partially reproduces the changes induced by traumatic injury. Gene expression alterations induced in spinal cord tissue by an indirect environmental stressor like a surgical operation may be also relevant for the development of tissue degeneration. This will be further discussed later in this paper.

### Gene expression analysis: data reproducibility

The reproducibility and reliability of our expression data were tested using the BeadStudio scatter plot analysis function to calculate the correlation coefficient (R^2^) of our gene expression profiles between technical replica, between sham-operated and control spinal cord tissues and between the thoracic and lumbar regions dissected from the spinal cord of the same *SOD1 G93A *mutant rats. These correlation analyses should demonstrate a significant level of similarity between the gene expression profiles under investigation (expressed by R^2 ^close to the identity value of 1). The highest correlation coefficient of R^2^: 0.9872 was detected, as expected, in the technical replica. Scatter plot correlation analysis (Table 1) of the thoracic and lumbar spinal cord expression profiles at 10 weeks of age, at onset and at end-stage disease, yielded correlation coefficients of 0.944, 0.947 and 0.932 respectively, indicating that the Bead array analysis captured the expected similarity between adjacent segments of the same tissue in the *SOD1 G93A *mutant rat. However, the degree of correlation diminished at end-stage disease, compared to the pre-symptomatic and the disease-onset stages. While the two segments of the same tissue remain overall very similar on a molecular level at a specific time point (as demonstrated by the high R^2 ^values), their molecular profiles diverge at the end-stage disease, possibly as a result of the different regional effects that the disease process has in lumbar and thoracic cord segments in hindlimb onset mutant rats. The correlation analysis between sham-operated and wild-type 10-week old thoracic cord samples identified a R^2 ^of 0.962 at 4 hours and of 0.973 at 30 minutes.

**Table 1:** Correlation analyses by scatter plot of Bead-array replica experiments (BeadStudio 3).

	G93A SOD1 10 weeks (L)	G93A SOD1 onset (L)	G93A SOD1 late-stage (L)	SCC 30 min.*	WT 10 weeks (T)
G93A SOD1 10-weeks (T)	0.944				
G93A SOD1 onset (T)		0.947			
G93A SOD1 late-stage (T)			0.932		
SCC 30 min.*				0.982	
Sham WT 30 min.					0.973
Sham WT 4 hours					0.962

Correlation coefficients (scatter plot analysis, BeadStudio 3) obtained comparing the thoracic to the lumbar spinal cord expression profiles from the same *G93A SOD1 *mutant spinal cord tissue at a pre-symptomatic stage, at onset and at end-stage disease (R^2^: 0.944, 0.947 and 0.932 respectively) and comparing sham-operated and naive thoracic cord samples from wild type littermates at 30 minutes (R^2^: 0.973) and at 4 hours (R^2^: 0.962) from surgery. A technical replica experiment is also reported, based on the comparison of the gene expression profiles obtained from the same spinal cord sample (extracted at 30 minutes from compression injury) used to hybridize two separate Bead arrays (R^2^: 0.9872*). The significant R^2 ^levels indicate that the Bead array technique reproduces the expected similarity of the samples chosen for the correlation analyses.

*: technical replica using RNA extracted from thoracic cord 30 minutes after injury (hybridization of two arrays in the same Bead Chip). The R^2 ^is reported in bold.

L: lumbar

T: thoracic

In this study, we have confirmed that our results reproduce the findings of a previous investigation performed using a similar experimental platform of mild SCI and the same time points for spinal cord gene expression analysis in the post-injury phase [11]. The expression of two subsets of genes included in GO categories/functional headings that were respectively up-regulated and down-regulated in our spinal cord compression experiment, was retrieved from the gene expression dataset GDS339 published by DiGiovanni et al. in Gene Expression Omnibus (GEO) [11]. This dataset reports the gene expression analysis at the site of mild injury in rat spinal cord 30 minutes, 4 hours, 24 hours and 7 days after trauma. The first subset included 80 genes belonging to GO categories activated at the 4 and 24 hours time-points from injury in our experiments (Figure 2A and Supplementary material 2A; GO:0001525_angiogenesis, GO:0045896_regulation_of_transcription__mitotic, GO:0007155_cell_adhesion, GO:0007049_cell_cycle; GO:0000278_mitotic_cell_cycle, GO:0016477_cell_migration, GO:0042551_neuron_maturation; GO:0030154_cell_differentiation). The second subset included 51 genes that are part of the gene categories becoming down-regulated at 7 days from injury in our experiments (Figure 2D and Supplementary material 2D; GO:0007267_cell-cell_signaling; GO:0007268_synaptic_transmission; GO:0019226_transmission_of_nerve_impulse; GO:0005856_cytoskeleton; GO:0005886_plasma_membrane; GO:0031410_cytoplasmic_vesicle; GO:0045104_intermediate_filament_cytoskeleton_organization_and_biogenesis and GO:0006694_steroid_biosynthesis).

**Figure 4:**
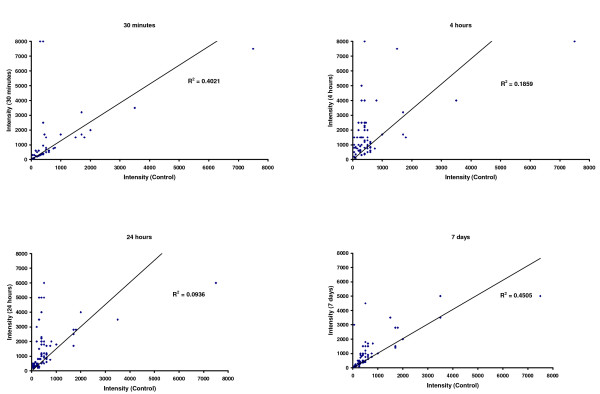
**Scatter plots showing the time-dependent up-regulation in spinal cord tissue at different time points after mild injury of a subset of 80 genes, according to the results of a published gene expression analysis from Di Giovanni et al.** [gene expression dataset: GEO-GDS339; [11]]. These genes belong to GO categories (e.g. GO:0001525_angiogenesis, GO:0045896_regulation_of_transcription__mitotic, adhesion, GO:0007155_cell_adhesion, GO:0007049_cell_cycle, GO:0000278_mitotic_cell_cycle, GO:0016477_cell_migration, GO:0042551_neuron_maturation and GO:0030154_cell_differentiation) that have been found, in our spinal cord compression experiments, to undergo the same pattern of differential regulation shown here in the DiGiovanni dataset at 4 and 24 hours after compression injury. Each gene expression value (intensity) was calculated as the average of 4 array experiments (GDS339) and represented in a scatter plot against the average value of 4 control array experiments [11]. The correlation analysis confirms that the findings of our gene expression analysis in compressed spinal cord reproduce previous observations obtained using a similar experimental paradigm of spinal cord injury.

The expression of the first subset of 80 genes taken from the data published by Di Giovanni et al. (2003) is shown in Figure 4. In each of the 4 plots, the gene expression values (intensity) are plotted against control array experiments and each plot shows a different time point after SCI. It can be seen that the gene expression of the test-genes increases significantly in contused spinal cord at 4 and 24 hours after injury (R^2^: 0.1859 and R2: 0.0936 respectively), compared to the 30 minutes and 7 days time points (R^2^: 0.4021 and R^2^: 0.4505 respectively). The correlation coefficients indicate more dispersion at 4 and 24 hours and the dots appear above the line of identity and close to the Y axis (Figure 4). These findings are in line with the substantial activation of the related gene categories/functional headings identified in our study at the same time points after compression injury (Figure 2A).

**Figure 5:**
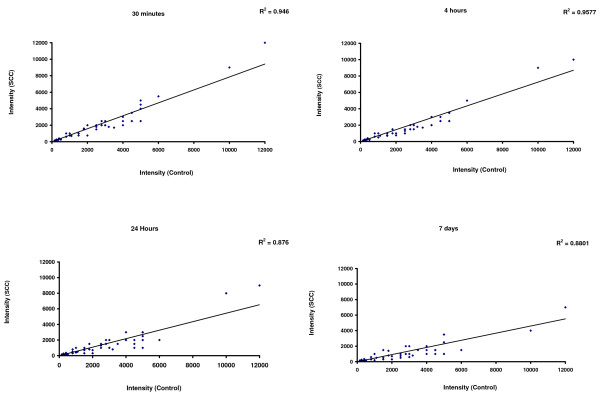
**Similarly to what shown in Figure 4, the scatter plots indicate the time-dependent down-regulation in spinal cord of 56 gene candidates identified in the experiment of mild compression injury published by DiGiovanni et al.** [gene expression dataset: GEO-GDS339; [11]]. These genes are included within GO categories and functional headings (e.g. GO:0007267_cell-cell_signaling, GO:0007268_synaptic_transmission, GO:0019226_transmission_of_nerve_impulse, GO:0005856_cytoskeleton; GO:0005886_plasma_membrane, GO:0031410_cytoplasmic_vesicle, GO:0045104_intermediate_filament_cytoskeleton_organization_and_biogene, GO:0006694_steroid_biosynthesis) that undergo down-regulation (particularly at 7 days after injury) in our spinal cord compression experiment. The pattern of differential regulation expressed as correlation coefficients (R^2^) in both subsets of up-regulated (Figure 4) and down-regulated genes according to the GDS339 expression dataset from Di Giovanni et al., correspond to the type and temporal pattern of differential regulation of the related GO categories and functional headings identified in our ontological analysis.

The expression of the second subset of 51 genes is reported in Figure 5. The expression of the test-genes is marginally reduced at 24 hours and 7 days from mild injury (R^2^: 0.876 and R2: 0.880 respectively), compared to the 30 minutes and 4 hours time points (R^2^: 0.946 and R2: 0.957 respectively). In the scatter plot analysis, dots representing the 51 genes are mostly below the identity line and close to the X axis at the 24 hours and 7 days time points (Figure 5). The gene expression data taken from DiGiovanni et al. [11] confirm the down-regulation of the related gene categories/functional headings at the same time points from compression injury in our study (Figure 2D).

Our study is primarily focused on the behaviour of molecular pathways across different experimental paradigms and not on the regulation of specific gene candidates. Therefore, we have not pursued any further confirmation of single transcripts differential regulation at RNA or protein levels.

### Common molecular features in trauma and genetically-induced spinal cord degeneration

Almost all differentially regulated GO categories in our study were found at 24 hours and 7 days post-SCC and at end-stage disease in the *SOD1 G93A *mutant spinal cord (Figure 2; see Additional files 1, 2, 3 and 4). In both animal models at these time-points and at an earlier stage after SCC, we have observed the up-regulation of structural and cytoskeletal genes and of factors involved in a variety of biological processes (Figure 2B; see Additional file 2). The structural genes which become up-regulated in both post-injury and in the *SOD1 G93A *mutant spinal cord, such as those encoding for proteins within microtubules, cell organelle and extracellular matrix may be crucial in the cascade of molecular events leading to neurodegeneration or may be important determinants in the induction of a condition like ALS following mechanical injury. It is now becoming increasingly clear that nervous tissue degeneration in ALS may derive from an inefficient or delayed clearance of different structural components, including neurofilaments [19]. In line with these observations, we have also identified an increase of protein folding activity induced by trauma and by the *SOD1 G93A *mutation (Figure 2B; "protein folding": 2 GO categories). We may postulate that trauma facilitates protein folding and the deposition of protein aggregates, which in turn may interfere with cellular functions such as axonal transport. The trauma-induced increase in structural protein content and aggregation may initiate a cascade of events leading to ALS. It is interesting to observe that in our gene ontology data, GO categories involved in microtubule formation appear to be activated also in sham-operated spinal cord at 4 hours, but not those categories that orchestrate protein folding. This finding may suggest that the mere production of neurofilaments in spinal cord tissue increases as a result of the stress induced by simple laminectomy and not necessarily following a direct traumatic injury. A recent retrospective and extensive epidemiological study on the incidence of ALS in a large area of Japan has demonstrated the relevance of environmental factors in the pathogenesis of ALS [20]. These include not only cervical spondylosis and spinal spondylotic myelopathy, but also a history of bone fracture and of surgical treatment.

A large-scale activation of gene categories involved in the modulation of apoptosis (10 GO categories) and of oxidoreductase activity (4 GO categories) appears to be a common denominator of the two experimental paradigms (Figure 2B; see Additional file 2). However, it is worth noticing that both types of response remain over-represented in the SOD1 G93A mutant spinal cord at a final disease stage, whereas this apoptotic signal is largely absent in the molecular profile of spinal cord compression at 7 days from injury. Spinal cord at 7 days after compression and in *SOD1 G93A *animals throughout the disease development appears to harbour a pattern of diffuse inflammatory responses, including activation of MHC, T-cell and complement factors. The early activation of factors involved in prostanoid metabolism in both experimental models is also in line with the surge of pro-inflammatory activity in spinal cord. A recent study has shown how astrocytes are directly involved in the complex regulation of proinflammatory prostanoids in the CNS, under stressful conditions induced by administration of lipopolysaccharide. Since astrogliosis is one of the main pathological features in the development of ALS and in the spinal cord response to injury, the activation of these pro-inflammatory factors may be a potential target of pharmachological intervention in both pathological conditions [21]. Altered brain prostanoid metabolism has already been implicated in ALS [22] and in the development of other neurological disorders including fronto-temporal dementia and Alzheimer's disease [23,24].

### Distinctive gene expression changes in spinal cord compression and G93A SOD1 mutant spinal cord: molecular and functional correlation

The ontological characterisation of the time-dependent gene expression changes in the spinal cord from the compression and *G93A SOD1 *animal models has also disclosed patterns of differential expression that are unique or predominantly represented in each experimental model. In this study, we have identified these distinctive molecular fingerprints and evaluated the related behavioural and functional changes in the animal model. In spinal cord subjected to compression injury, we have detected the up-regulation of a large array of growth factors included in 24 GO categories ("Development, growth" contains 24 GO categories, the largest number among all functional headings, Figure 3). Other gene categories including the bone morphogenetic proteins and factors involved in lipid metabolism and cholesterol transport which were found to become activated in our compression experiment are likely to have a similar pro-survival function. This powerful drive towards cell survival seems to be present from 4 hours after mechanical injury and to increase at the 7-day time point in the SCC experiment, whereas the *SOD1 G93A *mutant spinal cord appears to up-regulate growth factors only at disease onset. In SCC, this early surge of growth factors may counter the reported activation of apoptotic genes, which appears to reduce significantly after the 24-hour time point (Figure 2A/B; see Additional files 1, 2). The change in the relative balance between the expression of pro-survival and pro-apoptotic gene candidates may support the remarkable functional improvement in the animals subjected to SCC observed between 24 and 48 hours from compression (Figure 1C), whereas the persistently high level of expression of genes promoting apoptosis in the advanced stage of the disease in the *G93A SOD1 *mutants, coupled with a weaker and transitory activation of growth factors may explain the pattern of irreversible degeneration and functional decline in the transgenic animal.

During the highest level of functional recovery, between 24 and 48 hours after compression injury, we have also detected a spinal cord over-expression of GO categories that supervise cell cycle, mitotic transcription, adhesion, cell migration and taxis, angiogenesis and of *S100A*/Integrin/Polysaccharide binding (Figure 2A; see Additional file 1). The combination of these molecular responses appears to be unique to the trauma model (Figure 2A; 24 hours and 7 days time points after injury). It is possible that the majority of these molecular pathways are synergistic in maintaining or restoring tissue integrity, antagonising the effect of other cytotoxic molecular responses. For example, the gene candidates within the GO:0045896 category (_regulation_of_transcription__mitotic) are up-regulated at 4 and 24 hours from compression and are known to be involved in transcriptional regulation, increasing cell mitotic rate under conditions of injury [25]. The up-regulation of gene categories that promote cell migration/taxis, chemotaxis and phagocytosis (Figure 2A, see Additional file 1: 14 GO categories) is another distinctive feature of the effects of mechanical trauma in spinal cord. This is a chemoattractant-mediated inflammatory response which is known to lead to the release of nitric oxide (NO) and to be associated with secondary degeneration [26]. Our investigation shows how 9 GO categories representing different classes of chemokines and 8 GO categories of gene candidates involved in cell migration are significantly activated in all the time points considered after compression injury (Figure 2A; see Additional file 1), while the same chemokine pathways appear activated only at end-stage disease in the spinal cord from the *G93A SOD1 *gene mutant. These findings may suggest that any intervention of "vasculature remodelling" in spinal cord injury, enhancing blood flow and oxygen supply to the injury site may contribute to the functional improvement of damaged tissue. Another finding in keeping with the importance of angiogenic factors in SCC is the involvement of genes within the integrin-binding GO category. In our study, we have identified a dramatic down-regulation of this GO category at 30 minute from SCC coinciding with profound functional decline, followed by a 7-days significant over-expression of this gene category, while the animal model is in functional recovery. Integrins have been recognised as potential therapeutic targets to protect the vasculature and/or promote angiogenesis, as their activation can promote tubule formation and survival of endothelial cells in vitro [27]. Our experimental model of spinal cord compression seems to suggest that the activation of mechanisms facilitating tissue regeneration in injured spinal cord may be antagonised by the activation of cell-cycle pathways. At least 10 GO categories enlisting genes involved in different aspects of cell-cycle regulation become activated during the recovery phase following spinal cord compression (Figure 2A; see Additional file 1), whereas this molecular response does not seem to go above detection levels in the *G93A SOD1 *mutant spinal cord. Previous studies have shown how cell-cycle genes which become activated in both neuronal and glial cells following spinal cord injury cause neuronal and oligodendroglial apoptosis, glial scar formation and microglia activation (DiGiovanni et al. 2003). Treatment of SCI with the cell cycle inhibitor flavopiridol has been shown to improve functional recovery and to reduce lesion volume [28].

Another gene expression feature in the spinal cord from our trauma model which goes undetected in the *SOD1 G93A *mutant spinal cord is the remarkable down-regulation of a large array of genes involved in intracellular and in cell-cell signalling, along with gene factors crucial to ion and neurotransmitter transport and in synaptic structure (Figure 2D; see Additional file 4). The silencing of these important components of cell signalling at 7 days from compression injury is confirmed by our cross-analysis of the gene expression data from DiGiovanni et al. [11]. Recent experimental evidence has portrayed astrocytes as essential supporters of neuronal function and potential key players in the development of neurological and psychiatric disorders, for their ability to sense and integrate synaptic activity and to release gliotransmitters (e.g. glutamate, d-serine and ATP) [29]. Mechanical injury may specifically affect gliotransmission, causing the reduction of ion and neurotransmitter transport in the affected tissue. The ultimate effect on neuronal functional integrity of this generalised decline of cell-cell transmission in the traumatised cord at 7 days from impact is difficult to appreciate. It may be that the potential detrimental effect on cell survival of this phenomenon is counterbalanced by the reported activation of transcripts promoting cell growth and differentiation at this time point from injury.

#### Spinal cord degeneration and cytoskeletal changes

We have detected different patterns of expression of cytoskeletal and structural components in the two experimental models of spinal cord degeneration. In spinal cord after compression injury and throughout the disease development in *SOD1 G93A *mutant animals, factors involved in the formation of microtubule and cell organelles along with parts of the extra-cellular matrix appear to be variably up-regulated (Figure 2B; see Additional file 2). Our study shows also that cytoskeletal components such as actin and intermediate filaments, vesicle and synaptic proteins, muscle filaments and cell-matrix junction gene candidates become significantly down-regulated in spinal cord at 7 days after compression injury. We have confirmed that this down-regulation of structural genes in spinal cord has also been identified by another investigation employing a rat model of mild injury, at 7 days from the impact [[11]; Figure 5]. We could speculate that clearance of molecules that may potentially contribute to the formation of cell aggregates or be part of malfunctioning cell organelles, is part of a general process of functional restoration after traumatic injury. *SOD1 G93A *mutant spinal cord at end-stage disease does also show reduced expression of cytoskeletal genes, including intermediate neurofilaments which are essential for axonal integrity. The development of the disease in the *SOD1 G93A *model of ALS has recently been shown to result from damage to the distal motor axon and not only from activation of death pathways within the cell body [30]. The early involvement of axonal function and structure in conditions such as ALS has suggested that those axonal components that become deficient in the affected tissue at an early disease stage may be detected in biological fluids from ALS patients and used as biological markers of disease. Various studies have shown that slow destruction of neurons in most neurodegenerative disorders causes an elevation of neurofilament levels in the cerebrospinal fluid. Rosengren et al., found very high levels of neurofilament light chain in those patients with upper motor neuron involvement as opposed to lower motor neuron involvement alone [31]. CSF neurofilament heavy chain expression is also known to increase in proportion to the extent of upper motor neuron involvement and with disease severity [32].

Changes in the expression of actin and of those gene candidates involved in its metabolism appear as distinguishing features in the *G93A SOD1 *mutant spinal cord. At least 15 GO categories involved in actin protein polymerization become up-regulated at end-stage disease (Figure 2C; see Additional file 3). However, in both trauma and genetically-induced spinal cord degeneration it is possible to detect an early down-regulation of actin filaments including actinin, alpha 2 and 3 (*ACTN2, ACTN3*) and actin, alpha 1 (*ACTA1*) (GO:0005884_actin_filament Gene Category). The *SOD1 *mutated protein physically binds to actin, causing cytoskeletal and cell-cycle abnormalities with growth retardation, as recently shown by transfection with a human mutated *SOD1 *gene of neuroblastoma Neuro-2a cells [33].

These findings seem to support the hypothesis that spinal cord functional decline, whatever the initial pathological trigger, may be closely related to the abnormal homeostasis of a number of structural components, to an increase of protein folding and to the inability of the affected tissue of clearing aggregates of cytoskeletal proteins. In the *SOD1 G93A *model of ALS, the differential expression of various cytoskeletal and axonal proteins at different stages of the disease development makes the interpretation of the pathogenic role of these changes rather difficult. The down-regulation of actin, muscle and intermediate filaments at an early stage and throughout the disease process and the differential regulation of other cytoskeletal proteins central to the microtubule architecture towards the disease end-stage may be interpreted as one of the causative factors of neurodegeneration or simply be a consequence of other primary disease mechanisms such as slowing of axonal flow.

#### Inflammatory and immune responses in spinal cord degeneration

In both types of experimental spinal cord degeneration, we have identified inflammatory responses involving the activation of the MHC and complement complexes and of the innate and T-cell immunity (Figure 2B; see Additional file 2). A wealth of experimental data supports the concept that neuroinflammation in ALS spinal cord and cortex is based on an innate immune response regulated by macrophages and mast cells but also, on the contribute of an adaptive T-cell mediated immune response [34]. In line with our findings, the up-regulation of genes crucial to a T-cell-mediated inflammatory reaction has been already described in the spinal cord from patients with ALS. The ultimate effect on neurons and the interaction with activated microglia through secretion of cytokines that T-cells are supposed to exert is still a matter of intense investigation [35]. Whilst our study has clearly detected major analogies in the activation of inflammatory and immune factors in the spinal cord from two very different animal models of spinal cord degeneration, differences have also emerged which may help to characterise the specificity of each pathological process. For example, interleukine-6, TNF-alpha and other gene candidates supporting machrophage activation are significantly more expressed in injured spinal cord. This discrepancy may be related to the nature of the bioinformatic approach we have used, which focus on the significance of the activation of GO categories as entities (according to the level of enrichment of differentially regulated genes) rather than on the contribution and extent of differential regulation of single and potentially more crucial gene candidates.

The persistent activation of chemokines is another example of how an enhanced modulation of the inflammatory response is a characteristic feature of the spinal cord after compression (Figure 2A; see Additional file 1). The chemokines included in three GO categories in fact become up-regulated only at end-stage disease in the G93A SOD1 mutant spinal cord and with a lesser degree of statistical significance. The spatial and temporal distribution of proinflammatory cytokines and their cellular source has been previously investigated in a model of spinal cord injury [37]. Following mechanical trauma, it appears that all classes of neural cells initially contribute to inflammation, whereas recruited immune cells mostly determine its maintenance at later time points. With regard to the G93A SOD1 mutant spinal cord, our study suggests that the main inflammatory changes are present at end stage disease and are characterised by an adaptive T-cell mediated response, buffered by a modest increase of cytokine expression. These data on trauma and genetically-induced spinal cord degeneration may guide the timing and the nature of potential treatment strategies targeting the inflammatory response, once the effects on glial cells and neurons of these tissue responses are better understood.

## Conclusion

In this study, we have compared the gene expression changes characterising two models of spinal cord degeneration, using a bioinformatic tool that allows the simultaneous integration of expression data from different experimental sources, into categories containing ontologically comparable genes. The functional assessment of disease progression in the two animal models has enhanced our understanding of the significance of the dominant patterns of time-dependent gene expression changes. Our investigation seems to suggest that the remarkable functional improvement after mild spinal cord compression is sustained at a molecular level, by a powerful early onset surge of growth-promoting factors, possibly overriding the detrimental effect on cell survival that pro-apoptotic signals may exert. In contrast, the spinal cord molecular profile in the *SOD1 G93A *mutant rat undergoing functional decline appears to be dominated by pro-apoptotic and stress molecular responses, which are unchallenged by other major pro-survival signals. Our findings also support the role that neurofilament expression and aggregation may have in nervous tissue degeneration caused by different modalities of stress, as previously reported for ALS [37]. We could speculate that one of the mechanism involved in the induction of the irreversible neurodegeneration observed in ALS after mechanical trauma, could be the over-expression of structural genes in individuals with a genetically-determined inability of clearing these products in a way that would prevent the formation of cytotoxic cellular aggregates.

Our study shows how compression injury creates a particularly dynamic environment of molecular changes which may be pharmachologically modified for therapeutic purposes. Treatment strategies in SCI could rely on the blockade of factors involved in prostanoid metabolism and cell-cycle, or eventually, on the early enhancement of integrin and angiogenic factors. While we have concentrated in this study on a functional and molecular evaluation of the most represented gene expression changes in the experimental models, further investigations will be required to fine-tune our experimental approach to the relevance of single biological signals in the pathogenesis of spinal cord degeneration.

## Methods

### Animals

5 male Sprague-Dawley rats heterozygous for the G93A SOD1 gene mutation (Emerging Model 2148 Het Male, TACONIC US; Wyeth and Amyotrophic Lateral Sclerosis Association 2002) and 5 wild type females were used for a breeding project at Taconic Breeding Services (USA). An average of 5/6 pups per breeding pair were kept in separate cages and genotyped using a Multiplex Protocol for Human SOD (Taconic US). Male pups were discarded at weaning whereas a total of 20 heterozygous and 40 wild type females pups were kept in separate holding cages and shipped to our laboratories at 6 weeks of age. Following shipments, rats were housed in a pathogen-free animal facility at room temperature (21°C) under a 12 hour light-dark cycle. All animal procedures were conducted according to the Animals Scientific Procedures Act (1986), approved by the United Kingdom Home Office. 5 heterozygote female rats and 5 wild-type female littermates were sacrificed at 10 weeks of age and the thoracic/lumbar cord regions harvested for further analysis. Prior to sacrifice, the remaining rats were subjected to the behavioral and weight measurements as reported below.

### Laminectomy and spinal cord compression

Laminectomy with or without spinal cord compression (SCC) was performed in 24 wild type (WT) female rats at 10 weeks of age. Rats were anaesthetized in a fume box with a mixture of isoflurane (2.5%), oxygen and nitrous oxide (1:1 ratio) at a flow rate of 750–1000 mL/min. The skin and muscle surrounding spinal cord (T10 – L1) were incised and a laminectomy was performed at T12 level without damaging the dura. The compression injury was performed in 18 of the 24 rats by statically applying a 35 g. weight for 5 min, on a platform (area 2 × 5 mm^2^) resting on the exposed T12 spinal segment. In all animals subjected to surgery, the muscle layers were sutured and the skin was closed with wound clips. After surgery, animals were placed in warmed cages to recover from anaesthesia. Manual bladder expression was performed twice a day for the first week and once a week thereafter until establishment of self-voiding reflex. Rats were sacrificed by asphyxiation with carbon dioxide after surgery at the following time points: 5 rats 30 minutes after SCC and 3 rats 30 minutes after laminectomy, 4 rats 4 hours after SCC and 3 rats 4 hours after laminectomy, 4 rats 24 h after SCC and 5 rats 7 days after SCC. Spinal cord samples at T12 level were dissected after sacrifice and immediately frozen in liquid nitrogen.

### Behavioral analysis and weight measurements

The level of functional recovery in WT female rats after spinal cord compression (SCC) was scored at 4 hours, 24 hours and 7 days from injury using the Basso, Beattie, and Bresnahan (BBB) open-field locomotor rating scale as previously reported [18]. The BBB scoring for each animal right and left hindlimb was graded at the defined time points by two independent blinded observers.

The rat model of ALS carrying the *G93A SOD1 *gene mutation is known to develop subtle signs of motor impairment, with either hindlimb or forelimb distribution at disease presentation and to progress to end-stage disease in approximately 1 or 2 weeks from disease onset [38]. We have used body-weight measurements and behavioural analysis in 12 *SOD1 G93A *mutant rats with either hindlimb or forelimb onset and in 12 WT age-matched littermates as previously reported [11]. The rats were weighed using an electronic scale three times a week, from the age of 7 weeks to end-stage disease. In the *SOD1 G93A *mutant animal group, the maximum body weight reached by the animal before disease onset was set at 100%. The end-stage disease was defined as a reduction of 25–30% from the maximum body weight (normally this was accompanied by a consistent loss of the righting reflex; i.e. the failure of the animal to right itself within 30 seconds when placed on its side).

Of the 12 *SOD1 G93A *mutant rats used for weight measurements, 4 rats showing subtle signs of hindlimb paralysis at disease onset and 4 WT age-matched littermates were also subjected to serial evaluation of locomotor activity in an open field (80 × 30 × 30 cm polypropylene box), including the determination of the average number of rearing episodes and of the average immobility time. Each rat was video-recorded for 3 minutes three times a week. At disease onset (first signs of paralysis) and at end stage disease, average immobility time and average rearing episodes were recorded for each WT and *G93A SOD1 *mutant rat by independent investigators in two subsequent video clips.

The 4 WT and the 4 hindlimb-onset *G93A SOD1 *mutant rats subjected to behavioural analysis and weight measurements were sacrificed at end-stage disease. A further 4 *G93A SOD1 *mutant rats with early hindlimb paralysis were sacrificed at disease onset together with 4 WT age-matched littermates. Another group of 4 *G93A SOD1 *mutant rats and 4 WT age-matched littermates were sacrificed at a presymtomatic stage (10 weeks of age), when hindlimb or forelimb disease onset is clearly not predictable. Thoracic and lumbar regions of the spinal cord tissue were harvested and immediately frozen in liquid nitrogen. Spinal cord tissue from the WT rats at 10 weeks of age was also used as control group in the experiment comparing the gene expression profile in thoracic cord at different time points after compression with uninjured tissue.

### Bead array gene expression analysis

The RNA expression profiles of thoracic and lumbar spinal cord samples from the *SOD1 G93A *mutant rats and from WT littermates were obtained using large-scale gene expression analysis by Bead array Technology (Illumina, San Diego, USA). The same technique was employed to analyze the gene expression profile in thoracic spinal cord collected from WT female rats after mild spinal cord compression and after laminectomy. Spinal cord samples were collected for gene expression analysis from a total of 52 animals. RNA was extracted from the spinal cord tracts using the SV total RNA isolation system (Promega, UK) and quantified using a Nanodrop ND-1000 spectrophotometer. RNA quality was checked using the Agilent bioanalyser system (Agilent). RNA samples from the same experimental condition, genetic type (WT and *G93A SOD1 *mutant), time-point and spinal cord region were pooled together, for a total of 12 pools (4 pooled samples from the 30 min, 4 hours, 24 hours and 7 days time points after compression, 2 pooled samples from the 30 min and 4 hours time points after laminectomy, 3 pooled samples from pre-symptomatic, onset and end-stage *G93A SOD1 *mutant rats and 3 pooled samples from WT littermates at the same time points in the disease progression of the mutant rats). cRNA labeling was performed with 750 ng of total pooled RNA, using the Ambion Total Prep kit. cRNA purity and labelling was checked using the Nanodrop and Agilent bioanalyser. To ensure homogeneous conditions for the gene expression experiments, 500 ng from each of the 12 RNA pools were hybridised simultaneously to the 12 separate arrays contained in the same RatRef-12 Expression BeadChip, as per Illumina protocol (Illumina, San Diego, USA). A separate Beadchip was used to perform a technical replica experiment, testing the same pooled RNA sample with two separate arrays and to test pooled lumbar cord samples from *G93A SOD1 *mutant rats and from WT littermates, at 10 weeks of age, at onset and at end-stage disease.

Weighted averages of pixel intensities for each bead (local background was subtracted) and the relative p-value of detection were obtained through the workflow station (BeadArray Reader) and output files were analyzed using BeadStudio-3 (Illumina, San Diego, USA). Normalized expression data were obtained using the rank-invariant algorithm, to remove systematic variation of non-biological origin (BeadStudio-3). BeadStudio-3 was also used to test differential expression between RNA samples from the *G93A SOD1 *mutant and the WT rats and between RNA samples from injured/sham-operated animals and healthy rats at the chosen time points and spinal cord levels. Stringent data of differential expression were obtained using a false discovery rate through p-value adjustment. Fold enrichment values (experiment vs control) were used to obtain the list of candidates with greater than 1.5-fold change.

### Ontology analysis of the gene expression data

Text files containing lists of changed genes (i.e. genes that were up-regulated or down-regulated compared to controls) were prepared from the 9 sets of differentially expressed genes obtained using Bead array analysis as reported above. Each text file listed gene candidates symbols according to the rat genome database. As recommended by High-Throughput GoMiner , a flat +1 or -1 binary code was used to express up-regulation or down-regulation of genes in injured and *G93A SOD1 *mutant spinal cord (compared to healthy 10 weeks-old control tissue in the compression experiment and to the WT littermates in the *G93A SOD1 *mutant rats). The text files of changed genes were submitted electronically to the High-Throughput GoMiner database together with a list of all the probes contained in the Illumina rat chip. GO categories were identified and sorted according to a level of significance, based on a false discovery rate (FDR) threshold of 0.05 and on the level of gene enrichment. A multiple comparisons correction was used to eliminate GO categories that appeared significantly represented simply by chance. The analysis software generated significantly enriched GO categories with a FDR <0.05. It also provided integrative results, showing the FDR values for a selected GO category across all time points and experimental conditions.

Gene ontology data have been represented in the following way:

• We have grouped statistically significant down-regulated or up-regulated (FDR< 0.05) GO categories under a general functional heading (e.g. cell cycle, transcription, phagocytosis, angiogenesis etc.), considering the parent-child relationship of these GO categories and their functional similarity (most parent-child GO categories contain the same redundant sets of genes).

• Visual integration of the results was obtained using clustered image maps (CIMs) available from CIMminer and Excel drawing plattform and the results have been displayed as heat maps.

### Correlation analysis and cross-reference with other gene expression datasets

The reproducibility and reliability of the platform of gene expression analysis we have employed in this study was tested using the BeadStudio scatter plot analysis function to calculate the correlation coefficient (R2) of our gene expression profiles between technical replica, between sham-operated and control tissues and between thoracic and lumbar regions dissected from the same spinal cord samples.

A cross-reference analysis to confirm the reproducibility of our data was performed with the gene expression study performed at the site of mild injury at 30 min., 4 hours, 24 hours and 7 days from the trauma by DiGiovanni et al. [16]. Intensity values of two subsets of genes included in GO categories found to be up-regulated and down-regulated in a time-dependent manner after cord injury in our experiments were obtained from the dataset published by DiGiovanni et al. on Gene Expression Omnibus (GEO) [Di Giovanni et al. 2005] (GDS339; GEO Series: GSE464). The GDS339 expression data were obtained using an Affymetrix GeneChip Rat Genome U34 Array Set (RG-U34C, GEO GPL 85/86/87). Corresponding genes in the Illumina and Affymetrix gene chips were identified matching the different annotations to the same RefSeq Transcript IDs. Using the GEO "Gene profile" search-engine, we have calculated the average of the expression values from 4 SCI and 4 control arrays, for the corresponding genes in the datasets published by DiGiovanni et al. [16]. Average expression values for the test genes in the experiment from DiGiovanni et al. in injured and control spinal cord at each time point were reported as scatter plot and the correlation coefficient calculated.

## Abbreviations

*SOD1 gene*: Superoxide dismutase 1 gene; ALS: amyotrophic lateral sclerosis; MND: motor neuron disease; SCC: spinal cord compression; SCI: spinal cord injury; GO: gene ontology; GEO: Gene Expression Omnibus; BBB: Basso, Beattie, and Bresnahan; FDR: false discovery rate; aFDR: average false discovery rate; WT: wild type; *ACTA1*: Actin, alpha 1; *ACTN2*Actinin, alpha 2; *ACTN3*: Actinin, alpha 3.

## Authors' contributions

AM and JVP have drawn the experimental design of the study and elaborated the research strategy. AM has completed the database analysis, including the integration of expression datasets and the ontological characterisation of the gene expression changes using High-throughput GOminer. NJ and WLH have performed all the animal work and the related compression experiments, they have optimised all the methods of behavioural and functional analysis and obtained the gene expression data utilising the Bead array gene expression platform.

## Supplementary Material

Additional file 1 **GO categories and functional headings that are predominantly up-regulated in spinal cord after SCC.** The heat-chart reports those GO categories and related functional headings undergoing up-regulation that are computed by High-Throughput analysis only or predominantly in spinal cord after compression injury (Figure 2A). Click here for file

Additional file 2 **GO categories and functional headings that are up-regulated in spinal cord from *G93A SOD1 *mutant rats and from WT littermates subjected to compression injury.** The heat-chart reports those GO categories and related functional headings undergoing up-regulation that are computed by High-Throughput analysis in both spinal cord from *G93A SOD1 *mutant rats and in spinal cord from WT littermates subjected to compression injury (Figure 2B). Click here for file

Additional file 3 **GO categories and functional headings that are predominantly up-regulated in spinal cord from *G93A SOD1 *mutants.** The heat-chart reports those GO categories and related functional headings undergoing up-regulation that are computed by High-Throughput analysis only in spinal cord from *G93A SOD1 *mutants (Figure 2C). Click here for file

Additional file 4 **GO categories and functional headings that are down-regulated in spinal cord from *G93A SOD1 *mutant rats and from WT littermates subjected to compression injury.** the heat-chart reports those GO categories and related functional headings undergoing down-regulation that are computed by High-Throughput analysis in both spinal cord from *G93A SOD1 *mutant rats and in spinal cord from WT littermates subjected to compression injury (Figure 2D). S*: 30 minutes after sham operation. S**: 4 hours after sham operation. Pre: pre-symptomatic stage. Onset: disease onset. End: end-stage disease. Click here for file
